# Multicentric Castleman Disease of the Supraglottis: A Surgeons’ Dilemma

**DOI:** 10.22038/IJORL.2023.57806.2995

**Published:** 2023-03

**Authors:** Anna Fariza Jumaat, Mohd Razif Mohamad Yunus, Doh Jeing Yong, Reena Rahayu Md Zin, Marina Mat Baki

**Affiliations:** 1 *Department of Otorhinolaryngology Head and Neck Surgery, Faculty of Medicine, University Kebangsaan Malaysia Medical Centre, 56000 Kuala Lumpur, Malaysia.*; 2 *Department of Pathology, Faculty of Medicine, Universiti Kebangsaan Malaysia Medical Center, 56000 Kuala Lumpur, Malaysia*

**Keywords:** Castleman disease, Lymphoproliferative disorders, Neck, Plasma cells, Pharyngectomy

## Abstract

**Introduction:**

An abnormal mass in the head and neck involving the supraglottic and cervical region offers a wide range of differential diagnoses. The pathology is either benign or malignant in nature. Castleman disease (CD) is an uncommon lymphoproliferative disorder characterised by hypervascular lymphoid hyperplasia and is classified into unicentric or multicentric disease. Histopathologically it is divided into hyaline vascular (HV), plasma cell (PC), and mixed cellularity variants. The multicentric disease is linked with PC and has the propensity to progress to lymphoma or Kaposi Sarcoma.

**Case Report:**

We report a case of a 45-year-old gentleman who presented with a painless anterior neck swelling and left supraglottic mass for six months. Computed tomography (CT) contrast imaging demonstrated a homogenous enhancing lesion at the left supraglottic and the midline of the anterior neck with erosive changes of the thyroid cartilage. A surgical resection of the anterior neck mass was performed. The diagnosis of Castleman disease plasma cell variant was made by histopathologic evaluation. The patient remained well post-resection.

**Conclusion:**

Supraglottic multicentric Castleman disease is the least expected diagnosis in this case. Unicentric disease is treated with surgery. However, limited studies are available in determining the effectiveness of surgery in multicentric diseases. The plasma cell variant requires a multidisciplinary and multimodal approach due to an inclination towards malignancy. Research is needed to determine the role of surgery in multicentric disease and to develop optimum guidelines for managing cases. To date, there is unsubstantial literature describing supraglottic multicentric disease.

## Introduction

Castleman disease (CD) is a rare benign lymphoproliferative disorder, primarily characterised by progressive lymph node enlargement with an estimated incidence of 15.9-19.1 cases per million person-years (1). It is also known as giant lymph node hyperplasia or angiofollicular lymph node hyperplasia.^1^ The aetiology and pathogenesis of CD are not entirely understood. It has been acknowledged that this lymphoproliferative disorder is caused by human herpes virus infection of the B-cell pool and the lymphovascular compartment of lymph nodes (2-5). Much interest has been garnered in the pathogenesis of CD due to the increased documented reports of its incidence, namely in patients with seropositive *human immunodeficiency virus* (HIV) infection, with an estimated 50% occurring in seronegative HIV cases (2).

The distribution of lymphadenopathy in CD can range from pure unicentric disease to either a unicentric disease with several foci, or to multicentric disease with an anatomical area of predominance, or truly multicentric disease involving both peripheral and central lymphadenopathy (2,6). Unicentric Castleman disease (UCD) is a localised disease accounting for approximately 70% of cases which affects nodes located in the mediastinum (86%), thorax and retroperitoneum (11%). UCD infrequently involves the head and neck (6%-20%), axillary (4%) and inguinal regions (7,8). Out of the cases in the head and neck region, 85% are located in the neck. While the remaining occurrences are confined to the salivary glands (1). It is uncommon for CD to manifest in primary organs but can involve the spleen and the parotid glands (9). The symptoms are generally absent or self-limiting, which can be attributable to the mass effects. Multicentric Castleman disease (MCD) involving the head and neck region is reported to occur in up to 15% to 20% of cases. It primarily affects the lymph nodes and clincally presents as lymphadenopathy. Associated nonspecific symptoms include pyrexia, weight loss and lethargy. Some may exhibit abnormal systemic or autoimmune conditions, such as hypochromic microcytic anaemia, hyperglo- bulinemia, hyperalbuminemia, nephrotic syndrome, and hepatosplenomegaly (10). An elevated erythrocyte sedimentation rate (ESR), increased C-reactive protein (CRP), and abnormal white blood cell counts may occur (8). *Human herpes virus-8 *(HHV-8) may play a role in the pathogenesis of MCD (11). 

We report a case of a patient presenting with a midline neck mass with no systemic symptoms. The initial differential diagnosis of a thyroglossal duct cyst was made. However, the neck mass histopathological examination (HPE) revealed otherwise. CD of the head and neck can be confused with other neoplasms due to its paucity of signs and symptoms. This case report discusses the variation of clinical presentation and the recommended management of CD of the head and neck in HIV-negative MCD. Our literature review shows a few published articles describing HIV-negative plasma cell (PC) type MCD involving the head and neck region. However, no reports are available reporting PC type MCD specifically related to the supraglottis.

## Case Report

**Fig 1:**
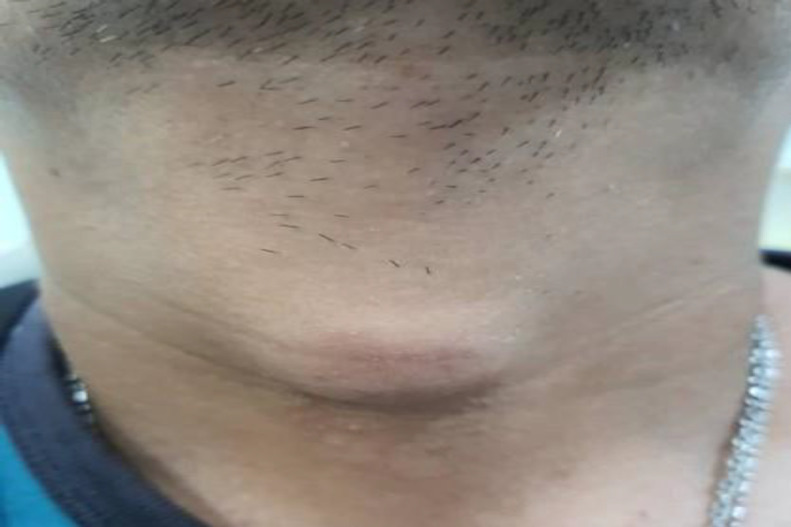
Anterior midline neck swelling measuring 3cm x 3 cm

A 45-year-old Chinese male presented with six months history of an anterior neck swelling that gradually enlarged over two months. It was painless and not associated with any disturbance in deglutition, breathing or speech production. There were no other related symptoms that were suggestive of infective aetiology. Physical examination revealed a 3cm-by-3cm anterior midline neck mass at the level of the thyroid gland (Fig. 1). 

**Fig 2:**
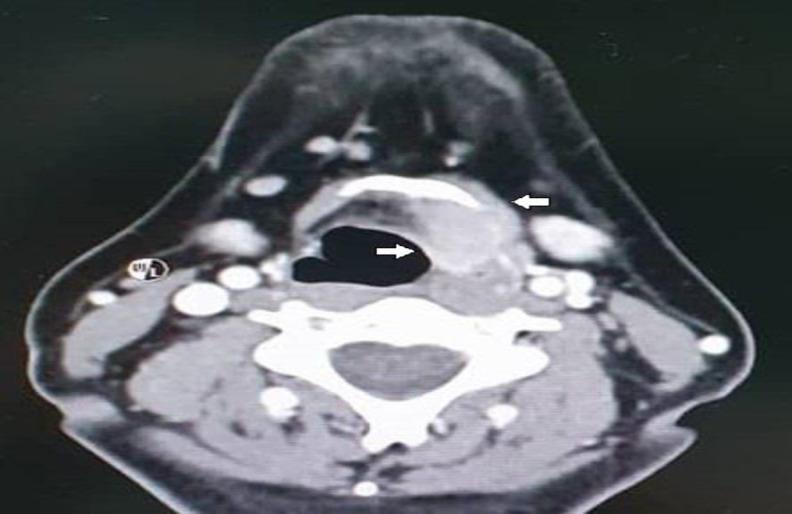
Computed tomographic scan contrast of the neck showing homogenously enhancing lesion at left supraglottic region measuring 2.3 x 1.7 x 2.5cm (white arrows). This lesion is causing widening of the paraglottic fat with medialisation of mucosal space

The mass moved with tongue protrusion and swallowing. A flexible fibreoptic nasolaryngoscopy revealed an obliterated left pyriform fossa. Fine needle aspiration cytology (FNAC) of the midline mass reported atypical mononucleated cells suggestive of neoplastic lesion. An ultrasound (US) guided tru cut biopsy was carried out to get further tissue for confirmation. Formal results reported back as reactive plasmacytosis. The contrast-enhanced computed tomography (CT) scan demonstrated a homogenously enhancing lesion at the left supraglottic region measuring 2.3cm x 1.7cm x 2.5cm, extending from the level of the hyoid bone to the level of the false cord inferiorly and posteriorly to the anterior commissure, causing widening of the paraglottic fat with medialisation of the mucosal space (Fig. 2).

**Fig 3:**
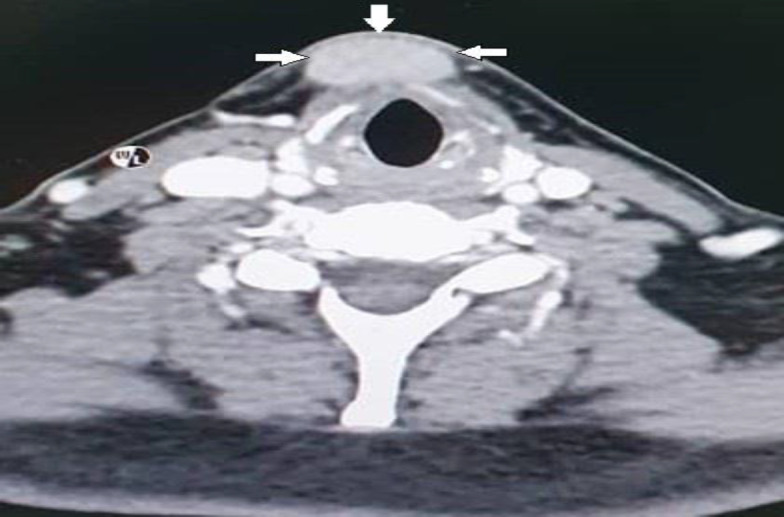
Computed tomographic scan contrast showing homogenously enhancing lesion at midline anterior neck at thyroid cartilage level measuring 1.3 x 2.5 x 2.1cm (white arrows). No clear plane with bilateral strap muscles. Features of erosion of the thyroid cartilage

The plane between the left strap muscles with this mass at hyoid and infrahyoid bone level is lost, displaying erosive changes involving the left thyroid cartilage (Fig. 3). 

The inferior aspect of the left aryepiglottic fold is thickened, obliterating the left piriform sinus. Another well-defined homogenously enhancing lesion at the anterior midline neck at thyroid cartilage level measures 1.3cm x 2.5cm x 2.1cm. Subcentimeter cervical lymph nodes bilaterally were present at levels Ia, Ib, II and III. There was no prominent lymphadenopathy seen in the mediastinum. 

**Fig 4:**
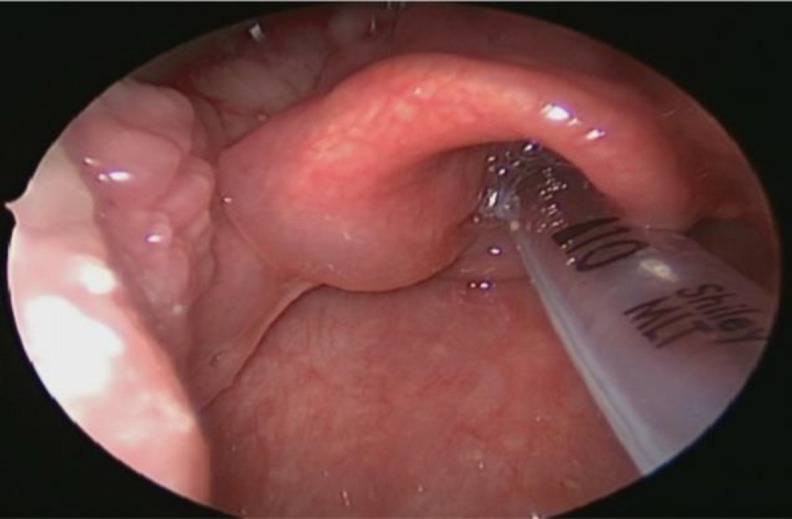
Direct laryngoscope view: The left epiglottis, lingual and laryngeal surfaces of epiglottis is edematous

He underwent a direct laryngoscopy (DL), anterior neck dissection and selective neck dissection. DL revealed an oedematous left epiglottis and arytenoids, and a biopsy was taken (Fig. 4). 

**Fig 5:**
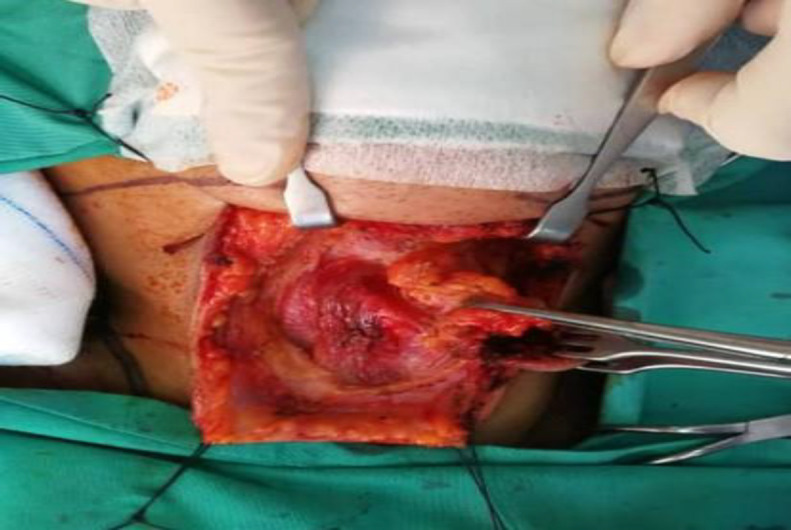
Anterior neck dissection specimen removed enbloc together with the lymphatic nodes

Intraoperatively, there was a mass at the anterior neck 4cm x 3cm below the skin (with skin tethering) and anterior to strap muscles. The anterior neck mass, fibrofatty tissue, and bilateral level 1b was removed en bloc (Figure 5).

**Fig 6:**
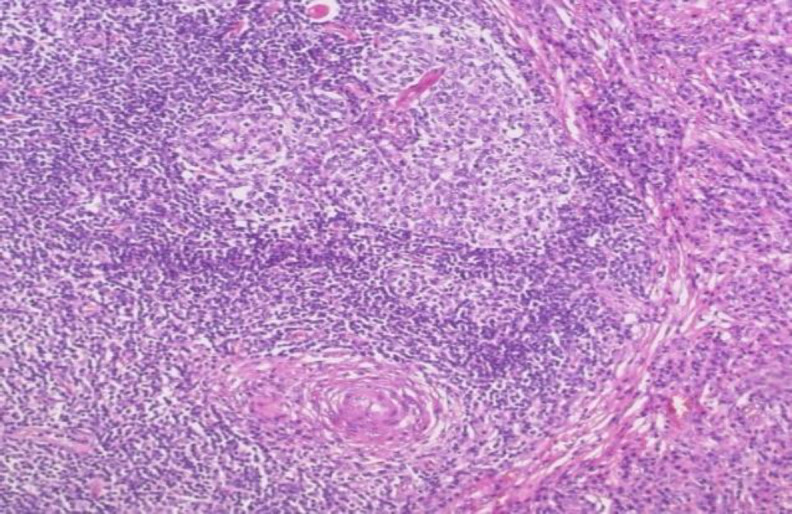
Involuted follicles with hyalinised blood vessels penetration. (x10 magnification)

Histopathology examination (HPE) of the supraglottic mass reported infiltration of deep subcutaneous tissue with dense lymphoid infiltrates composed of varying sizes of lymphoid follicles with hyperplastic germinal centres. The interfollicular area was expanded with numerous reactive plasma cells (Figure 6).

The Ki67 proliferative index was ~5%. The lymphoid cells are positive for cluster of differentiate 20 (CD20). The germinal centres are highlighted by CD 10. The follicular dendritic cell (FDC) meshwork is highlighted by CD21. 

He has recovered well after surgery and remains in remission on further follow-up. In addressing the remaining supraglottic mass, he was counselled for partial pharyngectomy for therapeutic purposes. However, the patient’s immense concern about the potentially associated surgical morbidities has hindered him from pursuing further completion of the resection of the supraglottic mass. He remains well today.

## Discussion

The clinical characteristic of this case demonstrates that reaching a final diagnosis has proven to be a challenge to the surgeon, despite obtaining FNAC and tru cut biopsy results. This is due to the non-specificity of the presenting symptoms with a midline neck mass that could lead to a misdiagnosis, such as a thyroglossal duct cyst or a thyroid nodule/cyst. The signs and symptoms in CD are atypical and rarely present in the head and neck region (12). CD has garnered much interest due to its rarity and association with HIV infection, HIV-negative patients and HHV-8 infection. CD is an uncommon disease with an estimated incidence rate of 15.9-19.1 cases per million person-years (13). Due to the paucity of studies, there is no appropriate management approach as our knowledge of the exact pathophysiology is limited, and more information is still yet to be discovered. Early literature has proposed a classification based on centricity, but additional classification has been implemented according to the histopathogenic type (3). 

Two main histopathology variants have been identified: hyaline vascular (HV) type (75-90%), plasma cell (PC) type (10-25%) and thirdly, variable degrees of mixed cellularity features in-between (15%) (2,7,11,14).

HV variant predominates in unicentric disease, accounting for approximately 75%-90% of patients (1,6). UCD is frequently associated with the HV variant, while the PC type is associated with MCD. Nevertheless, both histopathogenic types are predominantly associated with HIV-positive patients. There is a significant difference in the follicular dendritic cell network between HV disease and PC types. The dendritic network is typical in PC disease but is disrupted in HV disease with multiple tight collections of follicular dendritic cells (11). The HV variant displays prominent vessels with hyalinisation, and atrophic germinal centres with a characteristic concentric ‘onion-skin’ of mantle zone lymphocytes at germinal centres. These hyaline vessels enter the germinal centres to form a “lollipop sign” (8,12). The PC variant is distinguished by the significant presence of sheets of plasma cells in the interfollicular and perisinusoidal zones (8,11). The combination of the above two characteristics is considered mixed cellular variant. 

MCD is predominantly associated with HIV infection. The prevalence of MCD in HIV-positive patients is reported to be 8.3 cases/10,000 patient-years (15). The incidence of MCD in HIV-negative patients remains unknown due to sparse information and the availability of clinical studies (15). In the absence of HIV infection, UCD is the most frequent presentation, while HHV8 and HIV-positive patients invariably present with the multicentric disease (2,15).

MCD is linked to the aberrant human immune response and viral infection. HHV-8 is known to play a role in the pathogenesis and is detected in almost 100% of patients with seropositive HIV infection in MCD, as compared to 40-50% of MCD in patients with seronegative HIV (15,16). Patients with HHV8-positive MCD develop a more aggressive disease presentation with poorer prognosis. It has a median survival of fewer than thirty months, and the causes of mortality include sepsis, multiorgan failure, lymphoma, and Kaposi sarcoma (15,16). Its virulence is contributed by its ability to encode a viral-interleukin 6 (IL6), yielding increased levels of endogenous human IL-6.

The overproduction of the cytokine IL-6 causes plasma cell proliferation and aberrant IL-6 activity. This is exhibited by systemic symptoms, lymphoproliferation and the plasma cell dyscrasia of POEMS syndrome (polyneuropathy, organomegaly, endocrino- pathy, monoclonal protein, skin changes). These features are specific in patients with the PC type MCD (15,16). 

The disease presentation in HIV-negative patients can be categorised as either HV type UCD or PC type MCD. In a systemic analysis by Talat et al. and Taiqin et al., the authors identified characteristics associated with PC type MCD. The age distribution presentation of PC type MCD is between the fifth and sixth decade of life, with higher occurrences in male patients (9,12). For UCD, the most common histopathology type is HV, which is significantly associated with females within the third to fourth decades of life (12). Previous reports have contained variable information regarding systemic symptoms in MCD. Clinical studies have shown that patients with PC-MCD present with systemic B-symptoms; fever, fatigue, weight reduction, or night sweats (9,12).

Hepatosplenomegaly is present in most PC type MCD patients (Table 1). Abnormal laboratory results include leukocytopenia/ leucocytosis, anaemia, hypoalbuminemia, deranged coagulation values, increased CRP levels and lactate dehydrogenase (LDH) (9,12,15). In this case, the patient presented with atypical features of MCD, whereby his only complaint was a painless anterior neck mass with no other B-symptoms. Of note, he has normal blood parameters with negative results for HIV. However, he was not tested for HHV-8 infection.

A concise clinical workup is crucial in determining classification and staging. Pre-operative investigations can be challenging because FNAC and radiology studies are of limited value in concluding the diagnosis. CD can mimic other lymphoproliferative diseases and neoplastic disorders even when the differential diagnosis list is narrowed down. The patient should undergo a clinical examination of the peripheral lymph node that is most likely to be involved. Remotely located or visceral lymph nodes should also be investigated to exclude from CD. The best method would be a whole-body contrast CT scan directed at the neck, chest, abdomen, and pelvic regions (9). The primary purpose of imaging is to enable staging and distinguish between UCD and MCD. Defining surgical access and determining if the mass is resectable is vital. Hypervascularity generally indicates CD, but other differentials need to be ruled out (9,11). 

Surgery is the gold-standard treatment of UCD with or without radiation therapy and has an overall survival rate of 95%. (9,15). The principal treatment of UCD is total resection of the primary node. It is therapeutic as well as diagnostic (17). Unsuccessful removal of the involved lymph node represents a significant predictor for poor outcome. The surgeon should intend for the total removal of the primary lesion or node with free resection margins. Consider loco-regional systemic lymphadenectomy if a cluster of lymph nodes is involved; this usually encompasses the visceral nodes of the thorax and mediastinum. The overall result is better in resections of nodes located more peripheral than visceral territories (8,9,15). In an isolated case, Lei Jiang et al. reported a case whereby a patient had to undergo recurrent neck mass excision, ultimately leading to radical neck dissection. Unfortunately, HPE reported follicular dendritic sarcoma, and the patient perished due to metastases (17). (Table 1).

Management of MCD is utterly different from UCD. The clinician is faced with an entirely different setting. The clinician's role is limited; first, it is to gain tissue for diagnostic purposes, and second, to debulk the dominant foci of multicentric disease when there is the presence of life-threatening complications such as vascular or airway compromise, massive organomegaly, or bowel obstruction (9). Bowne et al. have described that partial resection of a large mass of involved tissue has been shown to produce transient symptomatic improvement in some patients. Therefore, it was concluded that neither splenectomy nor resection of lymph nodes offers any advantage to patients with the multicentric disease (17). The benefits of tumour debulking and resection in multicentric CD still need to be revised (9).

Hence, a multimodal therapy is recommended, which includes debulking surgery or resection when indicated, combined with single or multi-agent chemotherapy, followed by antiviral therapy for disease control and monoclonal antibody therapies targeting CD20 or IL-6 (9,15). Rituximab monotherapy is the mainstay of MCD therapy, especially in HIV-positive MCD, with a response rate of 67% (18). It is recommended that rituximab monotherapy is reserved to treat patients with no evidence of organ failure and combine it with other chemotherapy agents in those with aggressive disease.^16^ Rituximab is known to exacerbate Kaposi Sarcoma (18). Cytotoxic chemotherapy includes oral etoposide, vinblastine, cyclophosphamide, cladribine, chlorambucil, and liposomal doxorubicin. Siltuximab and tocilizumab are monoclonal antibodies which target IL-6 and its receptor (IL-6R). The downfall of monoclonal antibodies is the drug's cost, availability, and potential toxicity (19). There is no outcome difference comparing PC and HV type in the patient group with unicentric or multicentric disease (9), but the progress of the disease is more aggressive in patients who are seropositive for HIV infection (9,20,21). In a systemic review by Talat et al., the authors reported 64 patients with HIV-negative PC type MCD. It was observed that 45.7% had a 3-year disease-free survival (DFS) rate (9). While Oksenhaendler et al. observed that the median survival rate was fourteen months in patients with HIV-positive PC type MCD (21). Associated diseases observed in HIV-negative MCD patients included POEMS syndrome and paraneoplastic pemphigus. Paraneoplastic pemphigus and the POEMS syndrome occurred at a rate of 1.3% and 1.8%, respectively (9). A 32% occurrence of lymphoma and Kaposi sarcoma is linked with HIV-negative PC type MCD but had more significant incidence in HIV-positive MCD group (9,17). 

In a retrospective study involving five HIV-positive PC type MCD, two patients were simultaneously diagnosed with Kaposi sarcoma, and another eventually developed Kaposi sarcoma (Table 1).

**Table 1:** Table comparing patient demographics, clinical presentation, subtype diagnosis, HIV serology status, treatment and outcome in patients with plasma cell type multicentric Castleman’s disease of the head and neck and peripheral lymph nodes

**Author**	**Age (years), Gender**	**Presentation**	**Subtype Diagnosis**	**HIV serology status**	**Treatment**	**Outcome**
Taiqin et al 2021^12^	Median age: 47.5 years old *N* Male: 17 *N* Female: 7	Clinical presentation of head and neck not specified Systemic symptoms - fever, fatigue, weight loss, pneumonia, hepatosplenomegaly, serous effusion, kidney dysfunction	PC type MCD in 11 patients HV type MCD in 13 patients	Negative	8 cases CHOP 6 cases R-CHOP 1 case TCP 1 case AHSCT 4 cases refused treatment	4 cases passed away (not specified which patients)
Lei Jiang et al 2011^16^	50, male 57, male	Bilateral cervical lymphadenopathy Splenomegaly Left cervical lymphadenopathy Splenomegaly	PC type MCD PC type MCD	Unknown Negative	Bilateral neck dissection Refused chemotherapy CHOP (cyclophosphamide, doxorubicin, vincristine, prednisone)	Lost to follow up Defaulted treatment Passed away
Bowne et al 1997^17^	52, Male	PLN (site not specified) Hepatosplenomegaly Systemic symptoms	PC type MCD	Negative	Chemotherapy - cyclophosphamide, vincristine, carmustine, prednisone Splenectomy	Passed away
Oksenhendler et al 1996^21^	47, Male 22, Male 45, Male 67, Male 31, Female	All 5 patients presented with : - PLN (site not specified) - Systemic symptoms - Hepatosplenomegaly	PC type MCD	Positive	Splenectomy, CP, VP-16 ABV Splenectomy ABV Unknown	Developed KS Passed away Simultaneously diagnosed with KS Passed away Passed away Simultaneously diagnosed with KS Passed away Unknown

PC : Plasma cell; HV : Hyaline vascular; UCD : unicentric Castleman’s disease; MC : mixed cellularity; MCD : multicentric Castleman’s disease; RND : radical neck dissection; HPE : histopathology examination; BLEC : Benign lymphoepithelial cysts; CHOP : cyclophosphamide, adriamycin, vin cristine and corticosteroid; R-CHOP : rituximab and CHOP; TCP : thalidomide, cyclophosphamide, prednisone; AHSCT : autologous hematopoietic stem cell transplantation; PLN : peripheral lymphadenopathy; CP : cyclophosphamide, VP-16 etoposide; ABV : Adriamycin, bleomycin, vinblastine; KS : Kaposi sarcoma

## Conclusion

CD seldom involves the supraglottic and neck region. A supraglottic mass and cervical lymphadenopathy warrant a vast list of differentials, including malignancy and lymphoproliferative diseases, and the least expected differential diagnosis, in this case, is MCD involving the supraglottis. 

Most cases are HV type UCD, with a significant incidence in HIV-positive cases. UCD and MCD are two different conditions with diverse patient characteristics, manifestations, variable responses to therapy and long-term outcome. Acknowledgement of the subtypes of CD determines the surgeons’ role and approach in the diagnosis and treatment strategy. Surgery is the gold standard for treating UCD, but in MCD, it is a whole different picture. The patient's refusal to resect the supraglottic mass leaves the surgeon in a dilemma. 

The main objective is to provide the best outcome for the patient. PC type MCD requires close observation with a multidisciplinary and multimodal approach to treatment, which might be managed with chemotherapy, as there is an inclination towards malignancy. Further research is needed to determine the aetiology and pathogenesis of CD, to enable the development of enhanced and novel therapeutic options for this rare disease.

*Author Contributorship Statement*

AFJ interviewed, reviewed clinical notes and data, and wrote and submitted the manuscript. MRMY initiated the case report, provided images, and proofread and edited the manuscript. DJY contributed to data collection, providing patient images and editing the manuscript. MMB proofread and edited the manuscript. RRMZ provided histology slides, given comments on the histopathology section and edited the manuscript. All authors have read and approved the final manuscript.
